# Eusocial workers must evolve before maternal control

**DOI:** 10.1073/pnas.2422269121

**Published:** 2024-12-18

**Authors:** Jack da Silva

**Affiliations:** ^a^Discipline of Molecular and Biomedical Science, School of Biological Sciences, University of Adelaide, Adelaide, SA 5005, Australia

Rees-Baylis et al. (1) explore the role of maternal manipulation in the evolution of eusociality with an individual-based simulation in which mothers may evolve control of resource allocation to offspring, which affects offspring size, and offspring may evolve body-size-dependent dispersal, with smaller offspring benefiting from remaining in the nest as workers. They show that with maternal control, eusociality may evolve in the absence of strict lifetime monogamy, which has been argued as necessary for the high genetic relatedness between workers and their siblings that facilitates the evolution of worker reproductive altruism (2). However, there is a serious flaw with the Rees-Baylis et al. model and with the logic of maternal control as the basis for the evolution of workers. In this model, maternal manipulation can evolve only if offspring have previously evolved body-size-dependent dispersal and alloparental behavior, otherwise the mother produces undersized offspring that disperse and underperform as breeders, which would obviously reduce her fitness. Worker behavior must evolve before the evolution of maternal manipulation. That is, the evolution of worker morphology and behavior are initially decoupled, but may become coupled after the evolution of worker behavior (3). This is an important distinction because if worker behavior evolves first, then kin selection for reproductive altruism, rather than selection on maternal behavior, explains the origin of eusociality.

In the Rees-Baylis et al. model, worker behavior evolves in accordance with the Seger (4) hypothesis. This hypothesis posits that first-brood males disperse and survive to mate with second-brood/generation females before they enter diapause, thereby selecting for a female-biased sex ratio in the second brood/generation, which allows first-brood females acting as workers to mainly raise full sisters in the second brood/generation, to which they are related by ¾. The Seger hypothesis was proposed as an attempt to resurrect the central role of haplodiploidy in explaining the evolution of eusociality (5). However, there is no empirical evidence that this mechanism contributes to the evolution of workers (6, 7). More importantly, this mechanism cannot explain the evolution of eusociality in carpenter bees (Xylocopinae), which have unmated adult diapause (8), and for which maternal control has been reported (9). Therefore, the result that worker behavior evolves in the absence of maternal control below a mating frequency of 1.3 is based on an unsupported mechanism.

Maternal manipulation of daughter size must have evolved after the evolution of worker behavior. Daughters may have first delayed their dispersal if conditions were not conducive to their independent breeding or if there was the possibility of usurping or inheriting the breeder role in their natal nest. This would have presented opportunities to adult daughters to help guard or raise siblings while they “waited” (10). Maternal coercion could evolve after the evolution of plastic worker behavior, thus permitting the evolution of polyandry.
